# Racial Differences in Care Quality Among Men With Newly Diagnosed Prostate Cancer

**DOI:** 10.1001/jamanetworkopen.2025.23038

**Published:** 2025-07-24

**Authors:** Dawson C. Hill, Samuel R. Kaufman, Christopher Dall, Paula Guro, Sarah Leick, Preeti Chachlani, Xiu Liu, Addison Shay, Mary Oerline, Rishi R. Sekar, Lindsey A. Herrel, Brent K. Hollenbeck, Vahakn B. Shahinian, Arnav Srivastava

**Affiliations:** 1 Dow Division of Health Services Research, Department of Urology, University of Michigan, Ann Arbor; 2 Department of Urology, Massachusetts General Hospital, Boston

## Abstract

This cross-sectional study examines racial disparities in care quality, including confirmatory testing and potential overtreatment, among Medicare beneficiaries with newly diagnosed prostate cancer.

## Introduction

Black men with prostate cancer have historically had lower health care utilization, which is believed to contribute to their higher observed mortality rates.^1,2^ However, the relationship between health care utilization and quality of prostate cancer care is nuanced. Confirmatory testing among younger, healthy men electing for active surveillance to detect occult high-grade disease is guideline-concordant care and an example of alignment between utilization and quality.^3^ Conversely, in older, unhealthy men, utilization manifesting as immediate treatment (ie, potential overtreatment) often represents poor quality.^3^ In this scenario, treatment typically offers little oncologic benefit while posing substantial toxic effects. We examined racial differences in confirmatory testing and potential overtreatment—2 measures of quality with opposing relationships to health care utilization—in men with newly diagnosed prostate cancer.

## Methods

The University of Michigan Institutional Review Board deemed this study exempt because patient data were deidentified. We followed the STROBE reporting guidelines.

Using a 20% sample of traditional Medicare beneficiaries, we identified 54 979 men with newly diagnosed prostate cancer between 2014 and 2019 (analyzed in 2024). We created 2 separate study cohorts: men initiating active surveillance and unhealthy men at risk of overtreatment (eMethods in Supplement 1). The first outcome, confirmatory testing (ie, repeat prostate biopsy, magnetic resonance imaging before or after diagnosis, or genomics test within 12 months of diagnosis), was assessed among men initiating active surveillance.^4^ The second outcome, potential overtreatment, was assessed among unhealthy men (ie, those with >50% predicted noncancer mortality risk within 5 years of diagnosis). These men are highly unlikely to benefit from treatment (ie, surgery or radiation therapy) due to their competing health risks.^5^

Race, categorized using the Research Triangle Institute race code in the Medicare enrollment file (ie, Black, White, and other [not further specified]), was our main exposure. This designation demonstrates excellent agreement with self-reported Black race but might not fully capture its nature as a social construct.^2,6^ For patients categorized as other, further granularity about race was not available in the data. Adjusted odd ratios (aORs) were calculated using multilevel logistic regressions to evaluate the association between race and both outcomes (confirmatory testing and potential overtreatment), adjusting for age, socioeconomic status, Charlson Comorbidity Index score, rurality, and diagnosis year. From these models, we derived adjusted percentages for each outcome by race. Analyses were performed using Stata, version 18 (StataCorp LLC), with a 2-sided *P* < .05 considered significant.

## Results

Of 8051 men electing for active surveillance and 5090 unhealthy men at risk of overtreatment, 568 (7.1%) and 571 (11.2%) were Black, respectively (Table). Among men initiating active surveillance, Black race was associated with lower odds of undergoing confirmatory testing within 12 months of diagnosis compared with White race (aOR, 0.75; 95% CI, 0.62-0.92; *P* = .01). This finding translated to a 6.1% (95% CI, 1.9%-10.3%) decrease in confirmatory testing completion (Figure). Among unhealthy men at risk of overtreatment, Black race was associated with lower odds of overtreatment (aOR, 0.86; 95% CI, 0.77-0.97; *P* = .01) compared with White race. Overtreatment was less common in Black men compared with White men (adjusted difference, 6.1%; 95% CI, 1.8%-10.4%).

**Table.** Patient Demographics by Study Cohort

Characteristic	Men initiating active surveillance				Unhealthy men at risk of overtreatment			
	No. (%)			*P* value	No. (%)			*P* value
	Black	White	Other^a^		Black	White	Other	
No.	568	6804	679	NA	571	4173	346	NA
Age, mean (SD), y	69.5 (2.4)	69.8 (2.5)	69.1 (2.3)	<.001^b^	79.9 (5.0)	82.0 (4.9)	76.9 (7.2)	<.001^b^
CCI score								
0	303 (53.3)	4757 (69.9)	463 (68.2)	<.001^c^	27 (4.7)	317 (7.6)	127 (36.7)	<.001^c^
1	147 (25.9)	1252 (18.4)	122 (18.0)		67 (11.7)	538 (12.9)	55 (15.9)	
2	118 (20.8)	795 (11.7)	94 (13.8)		92 (16.1)	826 (19.8)	43 (12.4)	
≥3	NA	NA	NA		385 (67.4)	2492 (59.7)	121 (35.0)	
SES, tertile								
1	279 (49.1)	1737 (25.5)	126 (18.6)	<.001^c^	342 (59.9)	1578 (37.8)	67 (19.4)	<.001^c^
2	176 (31.0)	2450 (36.0)	215 (31.7)		153 (26.8)	1514 (36.3)	242 (69.9)	
3	113 (19.9)	2617 (38.5)	338 (49.8)		76 (13.3)	1081 (25.9)	37 (10.7)	
Rurality								
City	324 (57.0)	3122 (45.9)	381 (58.2)	<.001^c^	345 (60.4)	1763 (42.2)	76 (57.1)	<.001^c^
Metro county	166 (29.2)	2278 (33.5)	183 (27.9)		144 (25.2)	1470 (35.2)	39 (29.3)	
Near metro area or rural	78 (13.7)	1404 (20.6)	91 (13.4)		82 (14.4)	940 (22.5)	18 (5.2)	
Diagnosis year								
2014	71 (12.5)	782 (11.5)	46 (6.8)	<.001^c^	82 (14.4)	541 (13.0)	37 (10.7)	.24^c^
2015	82 (14.4)	977 (14.4)	73 (10.8)		70 (12.3)	591 (14.2)	38 (11.0)	
2016	79 (13.9)	1093 (16.1)	99 (14.6)		97 (17.0)	692 (16.6)	63 (18.2)	
2017	103 (18.1)	1260 (18.5)	137 (20.2)		100 (17.5)	711 (17.0)	52 (15.0)	
2018	104 (18.3)	1336 (19.6)	163 (24.0)		109 (19.1)	782 (18.7)	85 (24.6)	
2019	129 (22.7)	1356 (19.9)	161 (23.7)		113 (19.8)	856 (20.5)	71 (20.5)	

Abbreviations: CCI, Charlson Comorbidity Index; NA, not applicable; SES, socioeconomic status.

^a^ For patients categorized as other, further granularity about race was not available in the data.

^b^ Kruskal-Wallis test.

^c^ χ^2^ test.

**Figure.**
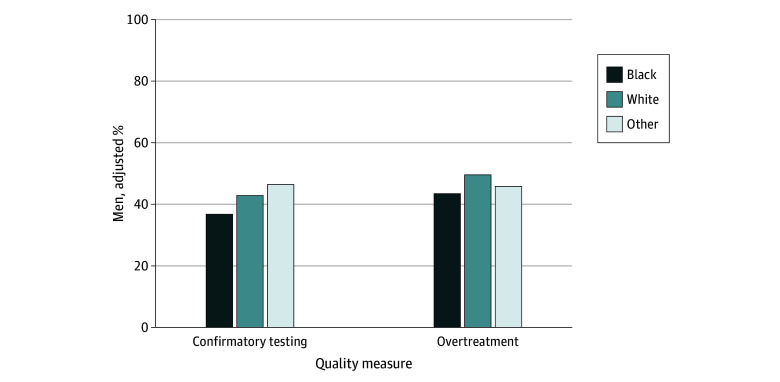
Adjusted Percentages of Quality Measure Adherence by Race Adjusted percentage for confirmatory testing for Black men statistically different than White men (*P* < .05). Adjusted percentage for overtreatment for Black and other men statistically different than White men (*P* < .05). Percentages were adjusted for socioeconomic status (tertiles), Charlson Comorbidity Index score, patient age, rurality, and diagnosis year. For patients categorized as other, further granularity about race was not available in the data.

## Discussion

Among men with traditional Medicare, we examined racial differences in 2 important measures of prostate cancer care quality that have contrasting relationships with utilization. Black men had lower odds of confirmatory testing among those on active surveillance, where utilization and quality are tightly aligned, indicating worse care. Conversely, Black men had lower odds of overtreatment, where utilization and quality are misaligned, suggesting better care in this dimension. Study limitations include the lack of cancer risk characteristics in Medicare claims, hindering our ability to classify appropriateness of management, and the smaller sample of Black men. Further, our use of a race variable might not fully address structural inequities faced by minority communities. Our study highlights the importance of understanding the nuanced relationship between utilization and prostate cancer care quality when trying to improve care for underserved communities.

## Notes

Supplement 1.
eMethods.

Supplement 2.
Data Sharing Statement
