# Supplementary material for: Racial Differences in Care Quality Among Men With Newly Diagnosed Prostate Cancer
Source: JAMA Netw Open. 2025 Jul 24;8(7):e2523038. doi: 10.1001/jamanetworkopen.2025.23038 (PMC12290725; doi:10.1001/jamanetworkopen.2025.23038)
Supplement: Supplement 1. — eMethods. [file jamanetwopen-e2523038-s001.pdf]

Supplemental Online Content

Hill DC, Kaufman SR, Dall C, et al. Racial differences in care quality among men with newly diagnosed prostate cancer. *JAMA Netw Open*. Published online July 24, 2025. doi:10.1001/jamanetworkopen.2025.23038

eMethods.

This supplemental material has been provided by the authors to give readers additional information about their work.

© 2025 Hill DC et al. *JAMA Network Open*.

eMethods.

Medicare population and creation of cohorts

We used a 20% national sample of men with Traditional Medicare to identify those with newly diagnosed prostate cancer between January 1, 2014, and December 31, 2019 (analysis performed in 2024). Men with newly diagnosed prostate cancer were identified using a validated algorithm with 99.8% specificity and 88.7% positive predictive value (n = 54,979).[1] Men included in our analysis had at least twelve months of follow-up and data available through December 31, 2020. We included only those men with continuous enrollment in Medicare Parts A and B. To establish comorbidities using the twelve-month period preceding the diagnosis, only those aged 66 and older were included, as entitlement for Medicare most commonly begins at 65 years. From this broader population of men with newly diagnosed prostate cancer, we created two study cohorts, men initiating active surveillance and unhealthy men at risk of overtreatment.

Men initiating active surveillance

We identified 8,051 men initiating active surveillance. These men—a relatively healthy subset of those undergoing conservative management, with a Charlson Comorbidity Index less than 3 and age less than 75 years — were identified using a validated claims-based algorithm that has a specificity of 97% and a negative predictive value of 92%.2 Among this cohort of men initiating active surveillance, we assessed receipt of at least one confirmatory test (i.e., repeat prostate biopsy, magnetic resonance imaging study, or genomic test) within 12 months of diagnosis, per clinical guidelines.3 Additionally, those with an MRI predating the diagnostic prostate biopsy were considered adherent.

Unhealthy men at risk of overtreatment

Given the protracted natural history of prostate cancer, clinical guidelines recommend against curative treatment in men with a high risk of noncancer mortality.[3, 4] We estimated each patient's probability of death from causes other than cancer (i.e., noncancer

© 2025 Hill DC et al. *JAMA Network Open*.

mortality) within five years using previously established methods.[5, 6, 7] Briefly, this model is based on a 5% sample of Medicare beneficiaries without cancer and models noncancer mortality using age, race, comorbidities, socioeconomic status, level of urban development (urban vs. rural), and geographic region. This mortality model has high discrimination (C-statistic = 0.90).[6] Using these methods, we identified 5,090 men with greater than 50% risk of noncancer mortality within five years and hence at risk for overtreatment. These men are unlikely to benefit from treatment (i.e., surgery or radiation therapy), even when facing higher risk tumors, due to their underlying competing health risks. We considered treatment in these unhealthy men as potential overtreatment, our second outcome.

Statistical analysis

Race was categorized using the Research Triangle Institute "race" code in the Medicare enrollment file and served as our main exposure (i.e., Black, White, or Other). While this designation is similar to prior literature, it may not completely capture self-identified race nor its nature as a social construct.[8, 9] However, prior work shows it has excellent agreement with self-reported Black race.[10] Additionally, further categorization for patients classified as "Other" was not available in our data.

Postulating that age, socioeconomic status, and level of urban development may have variable effects in Black and White men, we assessed three models using interaction terms between each of these variables and race. As the interaction terms were not statistically significant, they were not included in the final models. We also assessed our final models for multicollinearity by calculating variance inflation factor (VIF). When modelling confirmatory testing, the VIF equaled 2.6 and when modelling potential treatment, the VIF equaled 3.5, reassuring values that do not suggest any overt issues with multicollinearity in our regressions.

© 2025 Hill DC et al. *JAMA Network Open*.

eReferences:

[1] Hollenbeck BK, Bierlein MJ, Kaufman SR, et al. Implications of evolving delivery system reforms for prostate cancer care. *Am J Manag Care*. 2016;22(9):569-575.

[2] Modi PK, Kaufman SR, Qi J, et al. National Trends in Active Surveillance for Prostate Cancer: Validation of Medicare Claims-based Algorithms. *Urology*. 2018;120:96-102. doi:10.1016/j.urology.2018.06.037

[3] Schaeffer EM, Srinivas S, Adra N, et al. Prostate Cancer, Version 4.2023, NCCN Clinical Practice Guidelines in Oncology. *J Natl Compr Cancer Netw JNCCN*. 2023;21(10):1067-1096. doi:10.6004/jnccn.2023.0050

[4] Eastham JA, Auffenberg GB, Barocas DA, et al. Clinically Localized Prostate Cancer: AUA/ASTRO Guideline, Part II: Principles of Active Surveillance, Principles of Surgery, and Follow-Up. *J Urol*. 2022;208(1):19-25. doi:10.1097/JU.0000000000002758

[5] Gross CP, McAvay GJ, Krumholz HM, Paltiel AD, Bhasin D, Tinetti ME. The effect of age and chronic illness on life expectancy after a diagnosis of colorectal cancer: implications for screening. *Ann Intern Med*. 2006;145(9):646-653. doi:10.7326/0003-4819-145-9-200611070-00006

[6] Jacobs BL, Zhang Y, Schroeck FR, et al. Use of Advanced Treatment Technologies Among Men at Low Risk of Dying From Prostate Cancer. *JAMA*. 2013;309(24):2587-2595. doi:10.1001/jama.2013.6882

[7] Maganty A, Kaufman SR, Oerline MK, et al. National Trends in Management of Newly Diagnosed Prostate Cancer. *Clin Genitourin Cancer*. 2024;22(2):10-17. doi:10.1016/j.clgc.2023.07.001

[8] Dee EC, Todd R, Ng K, et al. Racial disparities in prostate cancer in the UK and the USA: similarities, differences and steps forwards. *Nat Rev Urol*. 2025;22(4):223-234. doi:10.1038/s41585-024-00948-x

[9] Vince RA, Eyrich NW, Mahal BA, Stensland K, Schaeffer EM, Spratt DE. Reporting of Racial Health Disparities Research: Are We Making Progress? *J Clin Oncol*. 2022;40(1):8-11. doi:10.1200/JCO.21.01780

[10] Jarrín OF, Nyandege AN, Grafova IB, Dong X, Lin H. Validity of Race and Ethnicity Codes in Medicare Administrative Data Compared With Gold-standard Self-reported Race Collected During Routine Home Health Care Visits. *Medical Care*. 2020;58(1):e1. doi:10.1097/MLR.0000000000001216

© 2025 Hill DC et al. *JAMA Network Open*.
