# Supplementary material for: Racial Differences in Care Quality Among Men With Newly Diagnosed Prostate Cancer
Source: JAMA Netw Open. 2025 Jul 24;8(7):e2523038. doi: 10.1001/jamanetworkopen.2025.23038 (PMC12290725; doi:10.1001/jamanetworkopen.2025.23038)
Supplement: Supplement 2. — Data Sharing Statement [file jamanetwopen-e2523038-s002.pdf]

Data Sharing Statement

Hill. Racial Differences in Care Quality Among Men With Newly Diagnosed Prostate Cancer. *JAMA Netw Open*. Published July 24, 2025. doi:10.1001/jamanetworkopen.2025.23038

Data
Data available: No

Additional Information
Explanation for why data not available: The data underlying this article were provided by the Centers for Medicare and Medicaid Services and cannot be shared by the authors of this manuscript under the data use agreement.
